# Electronic Free Energy Surface of the Nitrogen Dimer Using First-Principles Finite Temperature Electronic Structure Methods

**DOI:** 10.1021/acs.jpca.3c01741

**Published:** 2023-08-03

**Authors:** William Z. Van Benschoten, Hayley R. Petras, James J. Shepherd

**Affiliations:** † Department of Chemistry, University of Iowa, Iowa City, Iowa 52242, United States

## Abstract

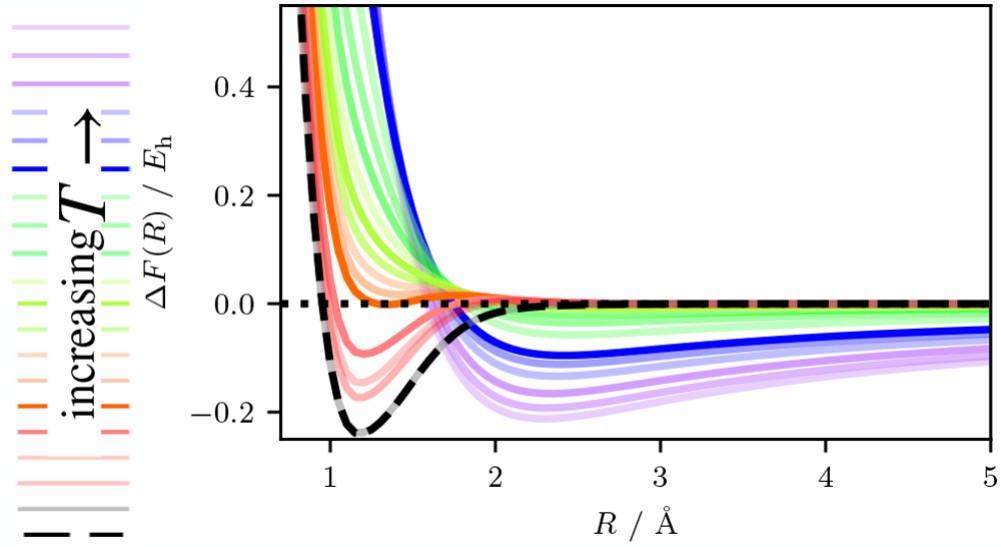

We use full configuration interaction and density matrix quantum Monte Carlo methods to calculate the electronic free energy surface of the nitrogen dimer within the free-energy Born–Oppenheimer approximation. As the temperature is raised from *T* = 0, we find a temperature regime in which the internal energy causes bond strengthening. At these temperatures, adding in the entropy contributions is required to cause the bond to gradually weaken with increasing temperature. We predict a thermally driven dissociation for the nitrogen dimer between 22,000 to 63,200 K depending on symmetries and basis set. Inclusion of more spatial and spin symmetries reduces the temperature required. The origin of these observations is explored using the structure of the density matrix at various temperatures and bond lengths.

## 1 Introduction

The Helmholtz free energy is an important thermodynamic quantity that can be used to describe chemical processes. A part of this is the electronic free energy and consideration of this quantity is important in solid state materials,^1−7^ spin-crossover systems,^8^ and in some cases, other molecular behavior.^9−22^ In the case of solids it is well-known that the electronic free energy alone can be a significant contribution in the accurate description for the material.^2−7^ On the other hand, the importance of electronic temperature in molecules has been demonstrated for specific situations such as spin-crossovers^8^ and at high temperatures.^11,13,17,19^ However, there is a need for more work to be done to fully understand the scope of how and when electronic temperature matters in molecules.^13^ One way to explore the scope is through *ab initio* electronic structure calculations.

Electronic structure calculations are performed at one state (ground or excited) when the temperature is zero. Some common methods to perform electronic structure calculations are density functional theory (DFT), with its many exchange–correlation functionals,^23^ and Hartree–Fock (HF) theory.^24^ DFT and HF methods are often used as starting points for more accurate methods for solving the Schrödinger equation, which come with additional computational expense. Some of these methods are coupled cluster theory,^25,26^ perturbation theory,^27,28^ configuration interaction,^29^ quantum Monte Carlo methods,^30−32^ and various numerical algorithms.^33,34^ Recently, there has been an eruption of method development targeted to finite temperature electronic structure. This includes methods such as coupled cluster theory,^35−42^ perturbation theories,^43−48^ as well as quantum Monte Carlo (QMC) methods such as density matrix QMC,^49−51^ Krylov full configuration interaction QMC,^52^ path integral Monte Carlo,^53−61^ auxiliary field QMC,^5,6,62−64^ and determinant Monte Carlo.^65^ There have been finite temperature formalisms for some time within density functional theory,^66−72^ Green’s function^7,73−81^ methods, and full configuration interaction^82,83^ methods.

The increased interest is in part due to the growing list of situations where finite temperature electrons are thought to play an important role in observed behavior. A nonexhaustive list of these situations includes Mott transitions,^84^ room temperature superconductivity,^85^ warm dense matter^86−92^ such as in planetary interiors or plasmas, metallic systems,^4,93^ phase diagrams of magnetic materials,^94^ ultracold atoms and molecules,^95^ thermally driven changes in structure,^96^ laser guided chemical reactions,^97^ spectral functions,^98^ heat capacities,^99^ and dielectric constants.^100^ One noteworthy example for our study involves the stretching of a periodic chain of hydrogen atoms.^5^ With the ever growing importance for understanding electronic behavior at finite temperature within the experimental and theoretical communities, now has never been a better time to contribute to this endeavor.

Our approach here is to undertake highly accurate finite temperature electronic structure calculations first using finite temperature full configuration interaction (ft-FCI)^47^ and then density matrix quantum Monte Carlo (DMQMC) when ft-FCI is too costly.^49^ The DMQMC method was originally developed and applied to the Heisenberg model and 1D spin systems. In doing so, the method was demonstrated to produce accurate finite temperature estimates of observables such as internal energies, staggered magnetization, and Renyi-2 entropy.^49^ Following the introduction of DMQMC, the interaction picture DMQMC (IP-DMQMC) method was developed.^50^ The IP-DMQMC method was then applied to the uniform electron gas, being used to calculate thermodynamic limit extrapolation using the static structure factor, and assisted in finding the finite temperature local density approximation functional with the help of the initiator approximation.^89,90,101^ Thereafter, four more developments were made through the efforts of our research: 1) The DMQMC and IP-DMQMC methods were extended to *ab initio* molecular systems.^102^ 2) The cost scaling for the method was investigated and found to scale like that of the ground state method full configuration interaction quantum Monte Carlo (FCIQMC) depending on the propagator symmetry.^30,103^ 3) The IP-DMQMC method was extended to sampling multiple temperatures thereby reducing the computational cost.^51^ 4) Gaussian process regression (GPR) was applied to the DMQMC data, and the electronic specific heat capacity and entropy were calculated.^104^ These are all important demonstrations of the viability of the DMQMC family of methods to treat the finite temperature electronic structure problem with high accuracy.

Our manuscript focuses on the investigation of Helmholtz free energy surfaces for the diatomic nitrogen molecule. We have performed analytical finite temperature full configuration interaction (ft-FCI) calculations, using a sum-over-states approach in the many-body basis for the N_2_/STO-3G dimer to calculate the free energy surface as a function of bond distance and temperature. This is done by first exploring the internal energy followed by the entropic contributions to the free energy surface. The behavior of the internal energy and entropy are related to each other before exploring the resulting free energy surface. A variety of temperature dependent features related to the minima and maxima found on the free energy surface are then investigated. Following this we explore what impact the symmetries used in the many-body basis have on the observed features for the free energy surface. As an important test for the conclusions made throughout the study, we go beyond the analytical ft-FCI calculations using the DMQMC for N_2_/cc-pVDZ. We close our study with an exploration of the density matrix structure and how it changes with temperature and bond distance for N_2_.

In this work, we will consider the diatomic nitrogen molecule in the canonical ensemble. For systems in which the particle number and charge will remain fixed, the canonical ensemble is required to accurately predict the behavior of these systems. One example noted in the literature is a case where Bose–Einstein condensates (BECs) of trapped ultracold atoms are being modelled.^105−107^ In general, the accuracy of the grand canonical ensemble and canonical ensemble are situation dependent;^107−109^ a paper by Shen et al. notes that molecules at finite temperature may be more accurately described by the canonical ensemble. On a practical note, DMQMC has only been developed for the canonical ensemble and this along with computer time cost are reasons to work in the canonical ensemble.^109^

## 2 Theory

### 2.1 Analytical Finite Temperature Electronic Structure

In the canonical ensemble we write the partition function as
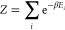
1where β = (*k*_B_*T*)^−1^ is the thermodynamic temperature and *E*_*i*_ are the energies corresponding to the eigenstates for our system. The eigenstates are found by solving the Schrödinger equation
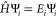
2where Ψ_*i*_ are wave functions for the eigenstates, and our electronic Hamiltonian is defined by
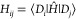
3where |*D*_*j*_⟩ are orthogonal Slater determinants. The full configuration interaction (FCI) method is used to calculate the complete set of eigenstates in the given basis.^47,82^

Using the partition function, we calculate the internal energy as
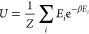
4the entropy as
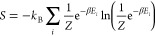
5and the Helmholtz free energy as
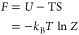
6We refer to these analytical sum-over-states formulas as finite temperature FCI (ft-FCI).

### 2.2 Free Energy Born–Oppenheimer

Throughout this section we will follow the notation found in ref (110). and derivation (with some notation) in ref (111). For a general quantum mechanical system consisting of nuclei and electrons, the wave function depends on coordinates of both types of particles. The Born–Oppenheimer approximation considers a wave function approximation that separates the total wave function into multiplicative terms of nuclear and electronic wave functions. This neglects terms that couple the nuclear and electronic degrees of freedom. The consequence of this is the ability to solve the electronic Schrödinger equation:
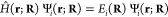
7where the electronic energy now only parametrically depends on the nuclear coordinates **R**. In this equation, **r** ≡ **r**^3*N*^ are the electronic degrees of freedom for the *N* electrons; **R** performs the same role for nuclei. The nuclear equation includes the electronic terms as an effective potential:
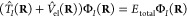
8where , where we have nonuniquely chosen *E*_0_ as the relevant electronic eigenstate, and this is more commonly referred to as the potential energy surface.

The free energy Born–Oppenheimer (FEBO) approximation^111−113^ is the finite temperature equivalent. Here, the free energies are calculated using the electronic eigenstates as
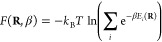
9This modifies the nuclear equation to be
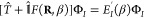
10and the total free energy can then be calculated as
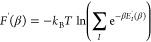
11which implicitly includes both the electronic excitations and the ionic vibrations through *E*_*I*_^′^(β).

The main advantage of the FEBO approximation is that the electronic excitation contribution (*F*(**R**, β)) can be found independent of the ion’s vibrational component. In this way, the vibrational component can be sampled independently using Monte Carlo or molecular dynamics.^113^ The direct consequence most important for this study is that the contribution from the thermally occupied electronic excitations can, and is useful to, be independently studied with respect to the nuclear coordinates **R**. Though the FEBO motivates investigating *F*(**R**, β) as a function of **R**, the mathematical derivation justifying this approach is extensive. Furthermore, the FEBO approximation, like the standard BO approximation, is applicable only under certain criteria. A detailed mathematical derivation for the equations in this section, as well as discussion of applicability, can be found in ref (112) and ref (111).

### 2.3 Quantum Monte Carlo

Given the complete set of eigenstates which satisfy eq 7 for a fixed **R**, we write the *N*-electron density matrix as
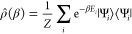
12where the wave function is defined as
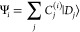
13Here *C*_*j*_^(*i*)^ are the wave function coefficients and |*D*_*j*_⟩ are the Slater determinants contained within the Hilbert space for a given number of electrons and set of one-electron orbitals. The density matrix is useful because it can be used to calculate finite temperature expectation values. For example the internal energy is calculated as
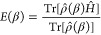
14

The use of eq 12 is contingent on solving the Schrödinger equation (eq 7) for all possible solutions given a fixed number of electrons *N* and one-electron orbitals *M*. This is possible for small numbers of electrons *N* < 10 and one-electron orbitals *M* < 20; however, for systems larger than this the computational cost quickly becomes impractical. To properly motivate the methods and procedures for solving eq 12, we follow the historical perspective for the development of DMQMC methods. We believe this gives a good understanding of the motivation for, and development of procedures used in, DMQMC. These originate from the ground state method full configuration interaction quantum Monte Carlo (FCIQMC).

#### 2.3.1 Full Configuration Interaction Quantum Monte Carlo

One way to overcome the cost barrier is by using Monte Carlo to solve the Schrödinger equation. FCIQMC solves for the ground eigenstate with energy *E*_0_ and corresponding wave function Ψ_0_.^30^ The FCIQMC method projects an initial condition to the ground state using the imaginary-time Schrödinger equation
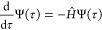
15Here, the exact ground state is found in the limit
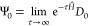
16where *D*_0_ is the initial condition. The efficiency of the FCIQMC method primarily comes from using a collection of signed (±1) particles, referred to as walkers, to represent the wave function. The walkers reside on the determinants |*D*_*j*_⟩ of the wave function, and the total magnitude or “population” *N*_*w*_(|*D*_*j*_⟩, τ) of walkers on a given determinant is proportional to that determinant’s coefficient *N*_*w*_(|*D*_*j*_⟩, τ) ∝ *C*_*j*_^(*i*)^. The use of walkers alone does not guarantee the efficiency of the FCIQMC method, but how they are evolved through imaginary-time does. In the first instance, the cost of memory is reduced by storing only determinants with a nonzero walker population in the computer memory. To take full advantage of this storage detail, any determinant with a population below one is removed or rounded in a nonbiased stochastic fashion. In the second instance, the cost of compute time is reduced by performing only a small number of the computations normally required by eq 15, which are stochastically selected on-the-fly for each walker. The stochastic noise is remedied with postcalculation analysis, where statistics from snapshots of Ψ_0_(τ) at large τ are used to calculate the exact-on-average energy *E*_0_.

#### 2.3.2 Density Matrix Quantum Monte Carlo

DMQMC is the finite temperature analogue of FCIQMC developed by Blunt and co-workers.^49^ In particular, the unnormalized density matrix written as
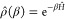
17can be solved using the symmetrized Bloch equation as its imaginary-time equation
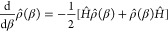
18given a known initial condition
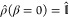
19The modifications required to represent ρ̂(β), rather than Ψ(τ), with a population of signed walkers are as follows. The first change is that walkers now reside on sites that are labeled using two Slater determinants |*D*_*i*_⟩⟨*D*_*j*_|. The site has a walker population *N*_*w*,*ij*_(β) which is proportional to the density matrix element; the label belongs to *N*_*w*,*ij*_(β) ∝ ρ_*ij*_(β). Just like in FCIQMC, only the sites with nonzero walker populations are kept in the computer memory. Additionally, like FCIQMC, the number of computations required to use eq 18 can be stochastically sampled. However, unlike FCIQMC, the selection of computations to carry out eq 18 is done twice as often. Once for the Slater determinant |*D*_*i*_⟩ and another time for ⟨*D*_*j*_|. The last change is in the postcalculation analysis. Where FCIQMC uses a single simulation to estimate the ground state energy *E*_0_, DMQMC samples the finite temperature energy *E*(β) for multiple β. For this reason, individual simulations termed a β-loop are repeated *N*_β_ times. These repeated simulations are then averaged over to produce the exact-on-average energy estimate *E*(β).

#### 2.3.3 Interaction Picture Density Matrix Quantum Monte Carlo

Following the development of DMQMC, Malone and co-workers made two important developments for DMQMC. The first development introduced the interaction picture to the DMQMC method. In the interaction picture, the Hamiltonian is varied through imaginary time rather than being constant. The simulation starts with an approximate Hamiltonian and ends with the exact Hamiltonian. The result is that a mean-field density matrix for the approximate Hamiltonian is used as the initial condition, which greatly improves the quality of statistics for a single targeted β.^50^ The second was the extension of the initiator approximation from FCIQMC to DMQMC, which helped alleviate the sign problem thereby reducing computational cost.^90,101^

In the interaction picture DMQMC (IP-DMQMC) method an intermediate matrix
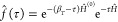
20is sampled rather than the density matrix (eq 17). The intermediate matrix connects an approximate density matrix to the fully correlated density matrix using the imaginary-time equation
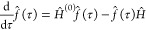
21given that the approximate density matrix is the known initial condition
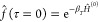
22In this work, the diagonal of *Ĥ* is used for *Ĥ*^(0)^. By evolving the initial condition through imaginary time τ, the fully correlated density matrix is sampled for a single β_*T*_, or the target β, which occurs only when τ = β_*T*_ and ρ̂(β_*T*_) are sampled. Since the approximate density matrix can take advantage of the energies along the diagonal of the Hamiltonian, the approximate density matrix tends to sample lower, and hence more important, energy states. In this way, the quality of the statistics improve. This comes with the caveat that repeated simulations can now be used to calculate *E*(β) for a single β = β_*T*_ only. Thus, multiple calculations are required to sample multiple β, which is not the case in DMQMC, which requires only a single calculation.

#### 2.3.4 The Initiator Approximation

The initiator approximation alleviates the numerical sign problem by restricting the walker dynamics to elements that are typically more important for the wave function or density matrix. In this way, the sign structure is stabilized and hence so are statistics accumulated from the wave function or density matrix. The downside is that accumulated statistics from the wave function or density matrix have a systematic error. Fortunately, the error is able to be systematically removed to an arbitrary degree.^90,101,114−118^

In the original initiator approximation for FCIQMC a new parameter *n*_add_ is introduced which modifies the Hamiltonian element *H*_*ij*_ depending on the walker populations residing on |*D*_*i*_⟩ and |*D*_*j*_⟩.^101^ Suppose determinant |*D*_*i*_⟩ with population *N*_*w*_(*i*) is attempting to contribute to determinant |*D*_*j*_⟩ with population *N*_*w*_(*j*) through eq 15. If both *N*_*w*_(*i*) and *N*_*w*_(*j*) are greater than zero, then Hamiltonian element *H*_*ij*_ is left unmodified. However, if *N*_*w*_(*j*) = 0 and *N*_*w*_(*i*) < *n*_add_, then the Hamiltonian element *H*_*ij*_ is set to zero. There is an exception to this rule: if a third determinant |*D*_*k*_⟩ is attempting to contribute to determinant |*D*_*j*_⟩ through eq 15 with the same sign as the contribution from determinant |*D*_*i*_⟩, the Hamiltonian element is left unmodified. The last change is if *N*_*w*_(i) ≥ *n*_add_ then*H*_*ij*_ is left unmodified. These rules were originally devised to reduce the impact of the numerical sign problem.^101^

These same rules are carried over to DMQMC; however, in place of the walker population *N*_*w*_(*i*), one considers the walker population on matrix element *N*_*w*_(*ij*) with a site label |*D*_*i*_⟩⟨*D*_*j*_|. This replacement is true for all determinants |*D*_*i*_⟩. Additionally, a second new parameter *n*_ex_ is introduced.^90^ The new parameter is used with the site labels |*D*_*i*_⟩⟨*D*_*j*_|. If the number of electronic excitations required to convert |*D*_*i*_⟩ to |*D*_*j*_⟩ is greater than *n*_ex_, then the Hamiltonian element will be set to zero. In cases where several criteria are met, the Hamiltonian element is left unmodified in priority of being set to zero.

#### 2.3.5 Piecewise Interaction Picture Density Matrix Quantum Monte Carlo

The DMQMC and IP-DMQMC methods were originally developed using model Hamiltonians,^49,50^ showing particular success in treating the warm dense electron gas.^90^ Several of us kept developing the DMQMC methods to treat *ab initio* systems.^102^ Most recently, some of us developed the piecewise interaction picture DMQMC (PIP-DMQMC) method.^51^ In PIP-DMQMC, the interaction picture propagator is generalized to sample temperatures beyond a target β (β_*T*_). The benefit of PIP-DMQMC is that the quality of statistics improves for a given simulation while also being able to sample a range of β ≥ β_*T*_ rather than only a single β = β_*T*_. In PIP-DMQMC, a piecewise matrix is sampled
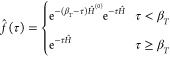
23with a corresponding piecewise imaginary-time equation
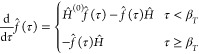
24which is used with the initial condition
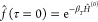
25Here we use the same *Ĥ*^(0)^ as in IP-DMQMC, the diagonal of *Ĥ*. We note that, in eq 24 above, we opted to use the asymmetric Bloch equation as the imaginary-time equation for τ ≥ β_*T*_. However, another valid imaginary-time equation is the symmetrized Bloch equation (eq 18). Our choice is based on minimizing the computational cost of running DMQMC methods, as asymmetric propagators have been found to have better computational scaling.^103^

### 2.4 Gaussian Process Regression

One of our latest developments was the application of Gaussian Process Regression (GPR) to data from the DMQMC family of methods.^104^ This allowed us to calculate the electronic specific heat capacity and entropy for systems beyond exact treatment.^104^ Here we provide a brief outline of the procedure required to calculate these quantities.

To calculate the free energy from PIP-DMQMC, we fit the internal energy using the GPy^119^ GPR library in a supervised fashion. The resulting GPR model is analytically differentiated and used to predict the specific heat capacity which is related to the internal energy by
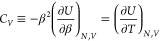
26and can be integrated numerically to calculate the entropy
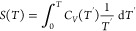
27where *T*′ is the temperature for integration on the bounds 0 to *T*. Finally, the original internal energy is combined with the entropy using eq 6 to calculate the free energy. The process is composed of the following steps:

1. PIP-DMQMC data, which are originally collected in intervals of *Δβ* = 0.001 from β_min_ to β_max_, are resampled to a user defined range β_r,min_ to β_r,max_ sampled in a user defined interval of *Δβ*_r_ = 0.1.
2. The data set resulting from step 1 has the ground state energy from FCIQMC added for a β twice as large as the β_max_ in the original PIP-DMQMC data set.
3. A model is trained on the β → *E*(β) data using a kernel comprised of the sum of the individual kernels: RBF, Matern32, Matern52, and the product between an RBF and Matern52 kernels.
4. The trained β model is used to predict the energy and specific heat capacity from β = 1.0 to β = β_max_ in steps of 0.01.
5. A trained *T* model, with predictions, is created by applying steps 1–4 using *T* = 1/β instead of β, and the FCIQMC energy is added at *T* = 0.
6. Supervised training is then used to adjust β_r,min_, β_r,max_, and *Δβ*_r_ for the β model and similarly for the *T* model such that the models produce a qualitatively reasonable specific heat capacity. The combined β and *T* subregions must span the original range of data from β_min_ to β_max_ for the original PIP-DMQMC data set.
7. Steps 1–6 are repeated several times, adjusting the β_r,min_, β_r,max_, and *Δβ*_r_ (and similarly the *T* equivalents) each time to improve the visually determined qualitative appearance of each model’s specific heat capacity.
8. The final β and *T* model predictions are combined to make a single data set which maps a single β (or *T*) to a single energy and specific heat capacity.
9. The combined β and *T* specific heat capacity is numerically integrated to calculate the entropy using eq 27, and the free energy is calculated with eq 6.

The final β_r,min_, β_r,max_, and *Δβ*_r_ values selected were bond length dependent. For *R* = 1.098 Å, β_r,min_ = 1.0, β_r,max_ = 25.0, and *Δβ*_r_ = 0.125. The *T* model was found to be sufficient and was used to predict from β = 1.0 to β = 25.0 in steps of *Δβ*_r_ = 0.01, while the β model was not used. For *R* = 5.49 Å, the β model used β_r,min_ = 1.0, β_r,max_ ≤ 10.0, and *Δβ*_r_ = 0.4. While the *T* model used β_r,min_ > 4.0, β_r,max_ = 50.0, and *Δβ*_r_ = 0.2. The β model predictions for β ≥ 1/0.205 were combined with *T* model predictions for β < 1/0.205. The final data set constructed from the combined predictions was from β = 1.0 to β = 100.0.

### 2.5 Free Energy Surface Calculation, Interpolation, and Analysis

The NumPy library is used to perform the sum-over-states required for equations in Section 2.1.^120^ For thermodynamic quantities calculated as a function of the atomic bond distance, cubic splines were used to fill in the data points between the bond distances. The SciPy library was used to perform the cubic spline interpolation, and the resulting spline was used to generate data from 0.8 to 100 Å in steps of 0.05 Å.^121^

For analysis on the potential energy surfaces, we considered only absolute energy differences ≥0.1 millihartree from the dissociation product (100 Å) as significant. The minimum and maximum contained in the energy surfaces were numerically identified by looping through each point in the data set (excluding the minimum and maximum bond lengths) and comparing the point to its two nearest neighbors. This process is done in order from the smallest bond length to the largest using the Numba library.^122^ If a data point is found to be smaller than its two nearest neighbors, we consider it a minimum; similarly, if it is larger, we consider it a maximum.

## 3 Results

### 3.1 Calculation Details

The FCI, FCIQMC, and PIP-DMQMC calculations were run with HANDE-QMC.^123^ The integrals required by HANDE-QMC were generated with Molpro^124^ using a restricted Hartree–Fock (HF) calculation. For generating and fitting N_2_/STO-3G^125^ thermodynamic quantities, integrals were generated for the bond lengths between 0.8 and 100.0 Å, with 100.0 Å taken to be the dissociation limit. All integrals for the main paper were generated using the frozen core approximation for the inner 1s electrons for each nitrogen resulting in *N* = 10 total electrons in the system. For comparing symmetries included in the Hamiltonian, we made two sets of integrals: one which had the orbital symmetry labels and one which did not. To restrict the FCI calculation to only the HF wave function symmetry from Molpro, we used the integrals which contain the orbital symmetry labels and provide the symmetry index from Molpro to HANDE-QMC. FCI calculations across all spatial symmetries used the integrals with no orbital symmetry labels. Finally, to perform FCI calculations across all possible spin and spatial symmetries, we manually looped through the valid pairs of α (*N*_↑_) and β (*N*_↓_) electrons which conserve the total electron number (*N* = *N*_↑_ + *N*_↓_).

For N_2_/STO-3G density matrix histograms, the bond lengths were (in Å): 1.098, 2.196, and 5.49. For all N_2_/cc-pVDZ^126^ calculations, the bond lengths were (in Å): 1.098 and 5.49. These distances were based on ref (115). The PIP-DMQMC calculations required the HF orbital energies which were manually calculated using the standard formula^24^ then added to the end of the integral file generated by Molpro.

In FCIQMC and PIP-DMQMC calculations, the reference Slater determinant was supplied to HANDE-QMC and the HF energy is provided in the data repository. Both FCIQMC and PIP-DMQMC used a simulation time step of *Δτ* = 0.001. The FCIQMC simulations used *N*_*w*_ = 10^7^ and *N*_*w*_ = 5 × 10^7^ walkers for the *R* = 1.098 and *R* = 5.49 Å simulations, respectively. PIP-DMQMC simulations were collected and averaged over *N*_β_ = 5 simulations using *N*_*w*_ = 5 × 10^8^ and *N*_*w*_ = 1 × 10^9^ walkers for *R* = 1.098 and *R* = 5.49 Å, respectively. Both FCIQMC and PIP-DMQMC used the initiator approximation with an initiator population of *n*_add_ = 3.0, and PIP-DMQMC also used an initiator level of *n*_ex_ = 2. In FCIQMC the simulation went ten time steps, and in PIP-DMQMC a single time step, before the shift was updated.^30,51^

### 3.2 Free Energy Surface Features

In this section, we discuss the effect of electronic temperature on the dissociation of N_2_/STO-3G using ft-FCI, all spatial symmetry blocks, and assuming no spin polarization. Our discussion focuses on five temperatures (0 K, 21,100 K, 31,600 K, 52,600 K, and 316,000 K) which highlight features that occur during heating of a diatomic bond, but we include a range of temperatures on figures to show the evolution of the dissociation with temperature. In each case, the temperatures correspond to a decimal number in Hartree atomic units, but we have converted the numbers to Kelvin and rounded to 3 s.f. for ease of interpretation.

**Figure 1:**
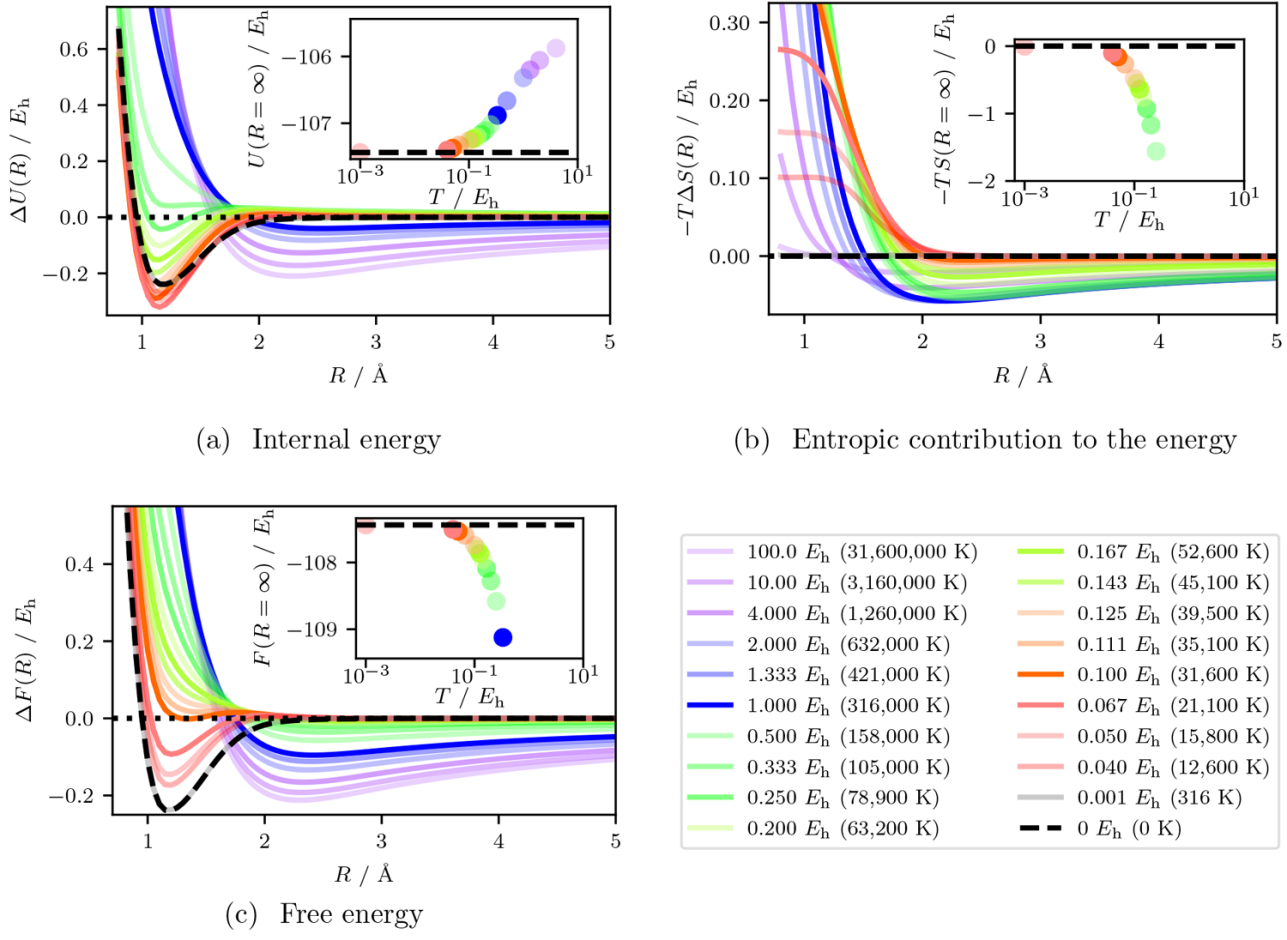
Analytical thermodynamic quantities as a function of bond distance over a range of temperatures for the N_2_/STO-3G system. The (a) internal energy and (b) negative of the thermally scaled entropy are added together to calculate (c) the free energy. Each temperature curve has the largest bond distance value subtracted, thereby setting the largest bond distance to zero. The values subtracted from each curve are shown in the inset for a small energy range near the ground state (*T* = 0 K). All temperatures are shown with a partial transparency except 0 K, 21,100 K, 31,600 K, 52,600 K, and 316,000 K which are left solid to aid with interpretation in the discussion. The analytical quantities are generated by using a cubic spline fit of each quantity as a function of several N_2_ bond distances. More details about this methodology can be found in Section 2.5.

Figure 1(a) shows the internal energy with the dissociated internal energy (*R* = 100 Å) subtracted from each temperature. The lowest temperature, 0 K, is the ground state dissociation energy of N_2_. This has a well depth of 0.2391 hartree which is also the dissociation energy measured from the bottom of the well, i.e., without vibrational zero-point energy (hereafter the dissociation energy). The internal energy at dissociation is shown in the inset.

As the temperature is raised, the internal energy of the bond lengths around the equilibrium geometry becomes lower relative to the dissociation energy, and this causes the well to become steeper. The bond length at which the energy minimum occurs also decreases. This effect is caused by the energy level spacing of the FCI eigenstates of dissociated products being closer than those states at the equilibrium geometry, resulting in the internal energy at dissociation increasing more rapidly than that at the equilibrium geometry. This occurs up to and including 21,100 K, where this effect reaches a maximum. Up to this point, in terms of the internal energy, the N_2_ bond becomes stronger.

Above 21,100 K, the well gradually becomes shallower, and the curve eventually gains a dissociation barrier. Then the curve becomes fully repulsive. Finally, at very high temperatures and energies, the internal energy develops a second minimum. The second minimum comes from the shape of the curve in the high temperature limit, which is equivalent to the average of the FCI eigenvalues and is basis set dependent.

Figure 1(b) shows the energetic contribution from the entropy over the same temperature range. At the ground state equilibrium geometry, the energetic contribution from the entropy mirrors the trend of the internal energy. For *T* > 21,100 K, the energetic contribution from the entropy rises to a maximum and then gradually decreases at higher temperatures. At longer bond lengths the contribution decreases and at higher temperatures there are some coordinates where the entropy contribution changes sign to stabilize an intermediate minimum. For the dissociation limit, the inset shows that we can observe a gradual decrease in the entropic contribution coming from the *T* term in the energy.

Figure 1(c) shows the free energy, which is the sum of the data in the two preceding graphs. As the temperature is increased from 0 K to 31,600 K, the well gradually becomes more and more shallow until the equilibrium geometry has the same energy as the molecule at dissociation. The temperature of 31,600 K therefore represents a temperature at which the bond does not (or barely) exists, i.e., a dissociation temperature. Such a dissociation point is reached due to a competition between the internal energy (which increases the dissociation energy) and the entropy (which decreases the dissociation energy). This draws attention to the importance of the entropy at elevated electronic temperature in providing qualitative features of the free energy surface. This, of course, has been observed in many different contexts.^4,127,128^ At the same time, a slight barrier (∼0.02 *E*_h_) is visible between the equilibrium bond distance and the larger bond distances. It is not until around 52,600 K that the bond does not exhibit a significant minimum in its free energy surface and is fully repulsive. This is a lower temperature than our calculated dissociation energy converted to temperature (*D*_e_ = 75,000 K). For *T* ≥ 316,000 K the second minimum has also appeared in the free energy and is the result of the average of the FCI eigenvalues as the entropic term becomes roughly constant. Observing the inset we see a decrease in the dissociated free energy with increasing temperature as it becomes dominated by the entropy.

**Figure 2:**
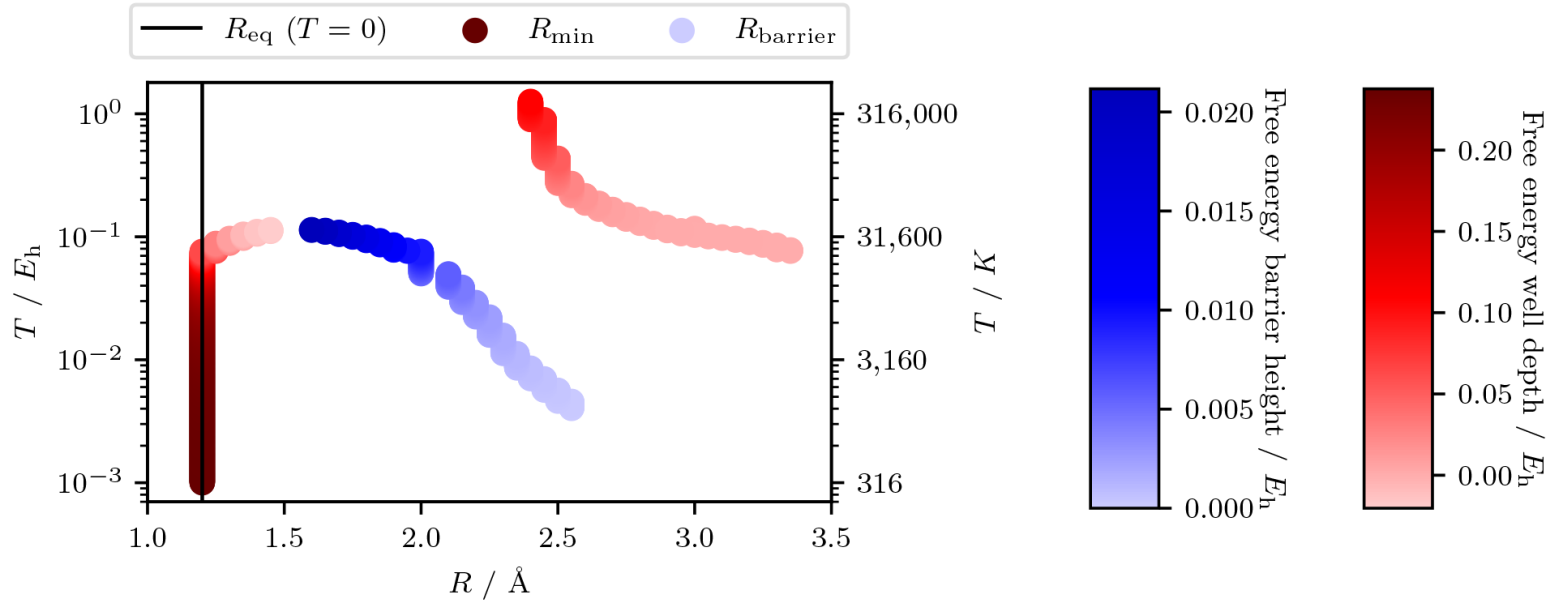
A plot showing the evolution of the free energy barrier and the free energy well depth as a function of both bond length and temperature for the N_2_/STO-3G dimer. The magnitude and sign of the free energy barrier and free energy well are described with a color-map shown on the right.

These findings are summarized in Figure 2. In this figure, the location and height (or depth) of the minima and maxima on the free energy surface are plotted as a function of interatomic distance and temperature. The color and darkness of the line represent the energy difference to dissociation. While the barriers are typically low for N_2_ and not enough to be significant at high temperatures, it is nonetheless of note that they are there in terms of the qualitative features of a free energy surface.

Starting with *T* = 0, the equilibrium bond distance is plotted (*R*_eq_ = 1.2 Å). At low temperatures, there is just one minimum on the free energy surface. As the temperature increases, this remains the case until approximately 600 K, where a barrier has emerged at *R* = 3.0 Å. As the temperature continues to rise, the well originating from *R*_eq_ remains unchanged, while the newly formed barrier moves to shorter bond lengths. After ∼22,000 K, the original minimum decreases its depth considerably, while the barrier height grows above 1 millihartree for the first time. At this point in temperature, we also observe the emergence of a second minimum for longer bond lengths. Then, at ∼30,000 K, both barrier and the first minimum are removed simultaneously, leaving only the second minimum; the second minimum begins to move to lower *R* as the temperature increases further. Eventually, the remaining free energy minimum tends towards approximately *R* = 2.4 Å with a depth of approximately 0.1 hartree.

Two further cases are included in the Supporting Information. We describe the impact of spin polarization on these graphs in Figure S1 and using a finite-temperature unrestricted Hartree–Fock (using a smearing approximation implemented in PySCF^129^) in Figure S2.

### 3.3 Thermal Dissociation Plots

**Figure 3:**
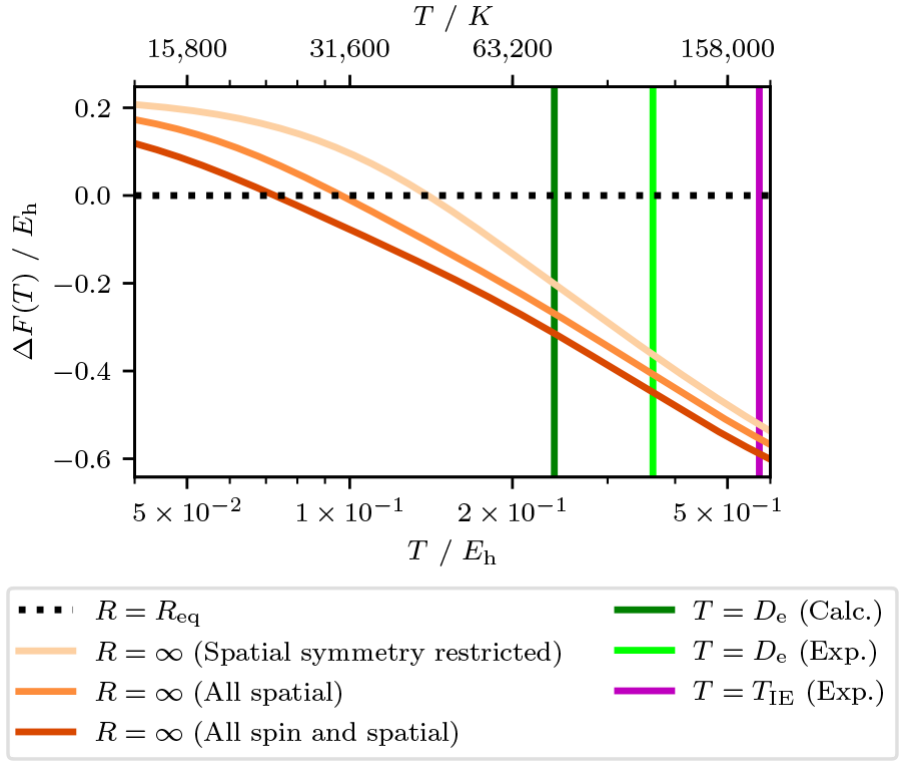
Free energy difference to the 1.2 Å separated N_2_ dimer (*R* = *R*_eq_) as a function of temperature for the N_2_/STO-3G system. Here *R* = ∞ refers to the largest bond distance in our data set (*R* = 100 Å). Where relevant, the symmetries for each calculation are indicated in the legend. For the spatial symmetry restricted (lightest orange) line, the ground state symmetry of the Hartree–Fock determinant was used. The calculated and experimental dissociation energy^130,131^ (*D*_e_) as well as the experimental first ionization energy^132^ (*T*_IE_) are shown to provide comparisons for interpretation.

Figure 3 shows a plot of the reaction free energy for dissociation found by subtracting the energy at the ground state equilibrium geometry from the dissociated limit. The change in sign represents the point at which the dissociated limit is more stable from the point of view of the free energy, i.e., when the nitrogen atoms cease to bond favorably, at 31,600 K. This is consistent with our observations from the dissociation energy graphs. We found no difference between this temperature and that calculated from the global minimum (instead of the ground state equilibrium geometry) and attribute this to how the low *T* minimum does not change very much as the temperature is raised (see Figure 2). This is because the minimum-energy geometry does not change significantly until after dissociation is energetically favored (Figure 2). The approach of using the ground state equilibrium bond distance has the benefit that only two geometries are being calculated. In general, it benefits our more expensive approaches if we can just use the ground state equilibrium bond distance even at the cost of a slight error, as our calculations span multiple temperatures at the same geometry. Sampling a range of temperatures similar to what is used in Figure 3 can cost hundreds of thousands of core hours, which restricts the number of calculations that can reasonably be completed due to cost and time.^51^

Also shown in Figure 3 are a number of different symmetry constraints being added or relaxed. Our estimate of 31,600 K for the crossover temperature assumes that all spatial symmetries are accessible in N_2_. When the Slater determinant basis of the calculation has its spatial symmetry restricted to that of the ground state Hartree–Fock determinant, the crossover temperature increases to *T* ∼ 45,000 K. Conversely, when states of all spin polarizations are added to the previously unpolarized calculation, the effect is the crossover temperature is decreased to 22,000 K. In this case removing (adding) eigenvalues means that the thermally driven effect has occurred at a higher (lower) temperature. This suggests that the eigenvalues being added or removed are qualitatively similar as a function of the bond distance. Overall, as more symmetries are included in the calculation, the crossover temperature decreases.

In the Supporting Information, we also consider how adding translational and rotational degrees of freedom can affect these results (Figure S3).

### 3.4 Results from QMC

**Figure 4:**
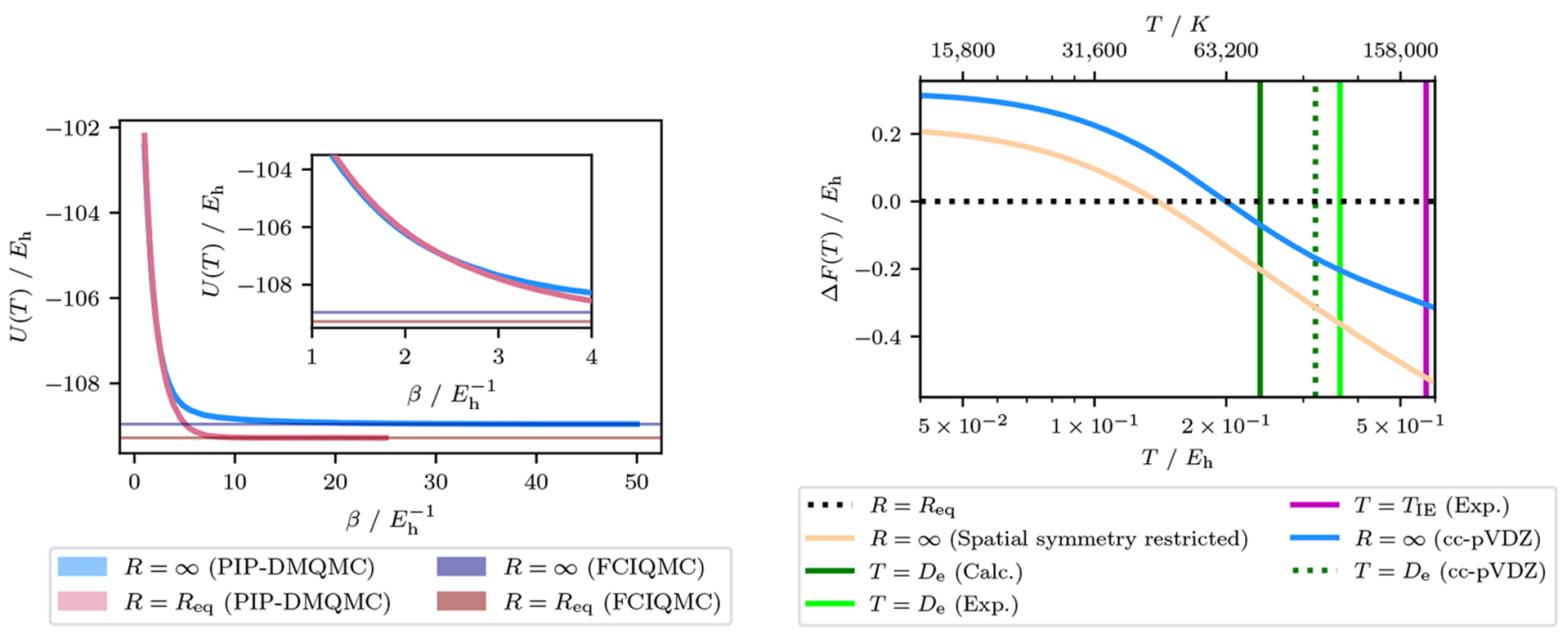
Thermodynamic quantities for two geometries of the N_2_/cc-pVDZ dimer, calculated with various methods. (a) The internal energy from the initiator PIP-DMQMC method using β_*T*_ = 1.0. (b) The data from Figure 1 with the free energy difference between the equilibrium and stretched geometry of the N_2_/cc-pVDZ dimer and the calculated dissociation energy from initiator FCIQMC added to the plot. N_2_/STO-3G data come from Figure 3. The shortest bond distance for N_2_/cc-pVDZ was used for *R* = *R*_eq_, and similarly, the longest bond distance was used for *R* = ∞, which can be found in Section 3.1.

To investigate the effect of basis set on the crossover temperature, we wanted to simulate a larger basis set, which requires quantum Monte Carlo. Here we 1) collected the internal energies for two N_2_ geometries in a cc-pVDZ basis using initiator PIP-DMQMC, 2) fit the internal energy using GPR and the fits were used to calculate the entropy, and 3) used the internal energy and entropy to calculate the free energy and compared the results to previous results. Figure 4 shows the PIP-DMQMC internal energies and the resulting free energies from this process.

Figure 4(a) shows the internal energies collected with PIP-DMQMC, as well as the corresponding ground state (*T* = 0) energies from the initiator FCIQMC for the two N_2_ geometries. We can observe that for both geometries the PIP-DMQMC internal energy converges toward the initiator FCIQMC energy as β increases, which is what we expected for well converged PIP-DMQMC data. An interesting feature of the stretched N_2_ PIP-DMQMC internal energies is the slower convergence to the ground state, with respect to β. This behavior is indirectly present in Figure 1(a) as the internal energy increases noticeably with a slight increase in temperature. This consistency is reassuring for the accuracy of both data sets and also serves as a visual explanation for why the internal energy changed more visually at larger bond distances in Figure 1(a) (with temperature).

GPR is fit to the PIP-DMQMC internal energies, and the GPR model is used to calculate the internal energy and entropy. The PIP-DMQMC+GPR entropic contribution is added to the PIP-DMQMC+GPR internal energy to produce the free energy (eq 6). The free energy difference for N_2_/cc-pVDZ is then added as a new line to Figure 3 which results in Figure 4. Here we only use the data for the spatial symmetry restricted in both STO-3G and cc-pVDZ. In addition to the PIP-DMQMC+GPR free energies, the reaction energy is calculated by using the FCIQMC data. Here we can observe similar features as before, namely there is a temperature (*T* ∼ 63,200 K) where the longer bond distance N_2_ has a lower free energy than the equilibrium N_2_ and hence is thermodynamically favored. This can be approximately considered the thermal dissociation temperature. To give context, the temperature for the sun varies from 5,800 K^133^ to 15,000,000 K^134^ and an average lightning strike is 30,000 K.^135^ Increasing the basis set led to the dissociation temperature increasing and becoming closer to not only the experimental value but also the calculated value. The thermal dissociation is closer to the calculated *D*_e_ value, even after considering the increased value in the calculated *D*_e_ which is a result of using a larger basis set. These results are promising as it has thus far demonstrated that the thermal dissociation energy appears to converge from below the experimental dissociation energy for N_2_. Furthermore, it demonstrates that the observations thus far are not primarily the consequence of basis set effects.

Going beyond a cc-pVDZ basis set and extrapolating to the complete basis set limit are important goals for this kind of work, but we felt that a detailed investigation for dimers was beyond the scope of this paper. Instead, we have provided a simple investigation of a smaller system, H_2_, in the Supporting Information section entitled “The complete basis set limit”.

### 3.5 State Histograms

Until now, we have investigated several interesting behaviors found in the free energy surface for N_2_. Here we will investigate some reasons for the behavior explored in the previous sections. To do this, we will use state histograms which were originally developed for FCIQMC.^115^ These histograms are collected by looping through the coefficients present in an FCIQMC simulation. These coefficients are based on a snapshot of the FCIQMC wave function. The population of each coefficient is compared to a set of bins (each with its own unique population range). If the coefficient’s population is greater than or equal to the bin’s population, the count of the bin is increased by one. For DMQMC, we have extended this process for analysis of the density matrix elements rather than coefficients of the wave function. The resulting plot shows the number of matrix elements with the corresponding number of normalized walkers, such that the largest relative population is one.

**Figure 5:**
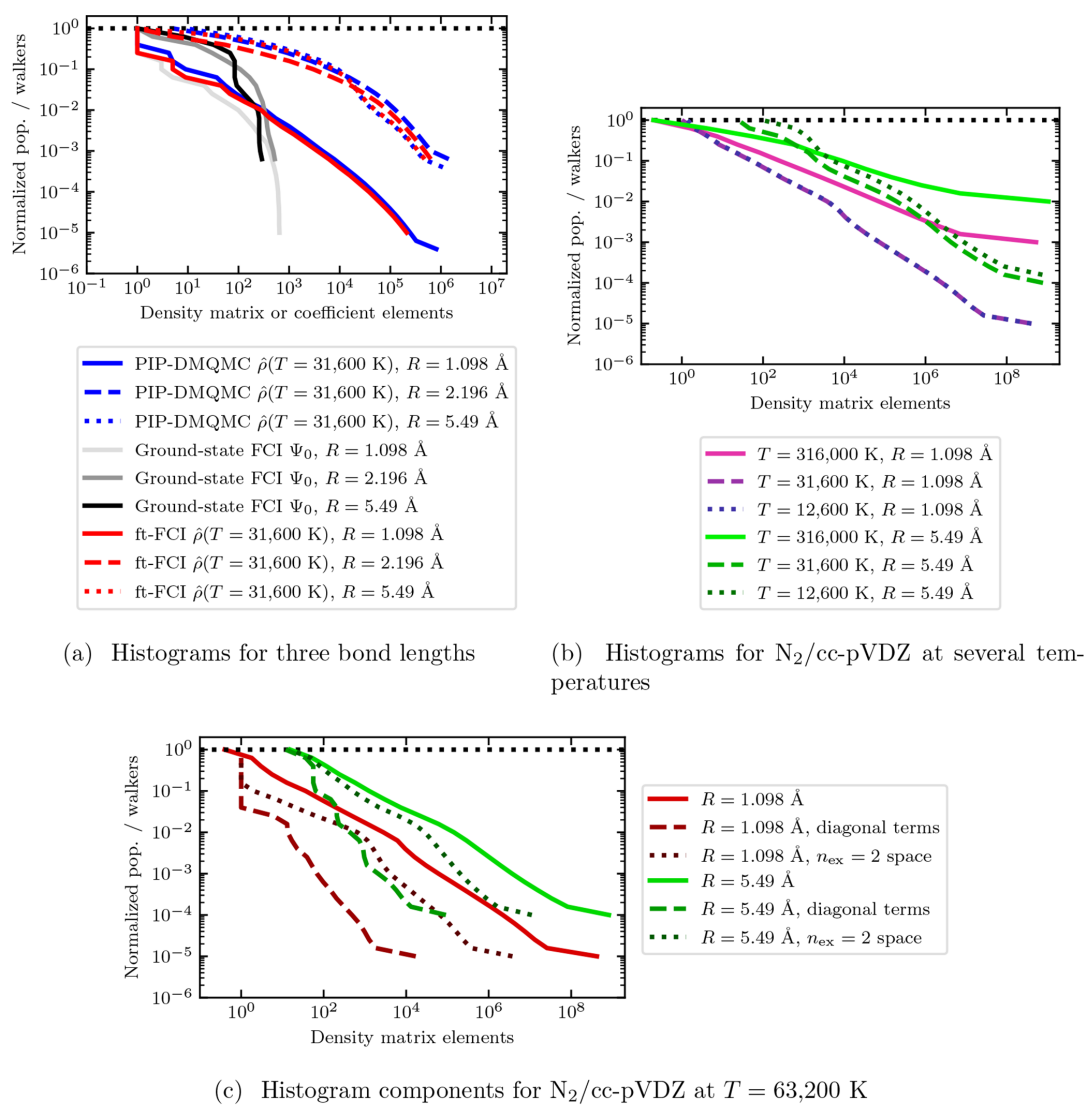
Walker population distribution histograms from several methods for a variety of bond lengths and temperatures. (a) The histograms for the ground state wave function from FCI and the density matrix from ft-FCI and PIP-DMQMC at *T* = 31,600 K for three bond distances. (b) *R* = 1.098 and *R* = 5.49 Å bond length density matrix histograms. (c) Histograms for some of the contributions to the density matrix at *T* = 63,200 K for N_2_/cc-pVDZ. Unless indicated otherwise, data correspond to N_2_/STO-3G. PIP-DMQMC data are averaged over *N*_β_ = 5 runs with *N*_*w*_ = 10^7^ for calculations using the STO-3G basis set and *N*_*w*_ = 5 × 10^8^ (*R* = 1.098 Å) or *N*_*w*_ = 10^9^ (*R* = 5.49 Å) for calculations using the cc-pVDZ basis set. All PIP-DMQMC simulations used the initiator approximation with an initiator level of two (*n*_ex_ = 2) and an initiator threshold of three (*n*_add_ = 3.0).

To familiarize readers with the types of information that is able to be extracted from state histograms, one line in Figure 5(a) shows the *R* = 1.098 Å bond length ground state FCI wave function histogram. Here it is observed that there is a single coefficient with a relative population of one. Thereafter, the relative population immediately falls roughly an order of magnitude before hitting a plateau and decreasing modestly as the number of coefficients increases. This behavior is consistent with little to no static correlation present in the system.^115^ As the number of coefficients is increased further still, the relative population quickly falls off toward the smallest relative population. Comparing the initial behavior of *R* = 1.098 Å to the longer bond lengths *R* = 2.196 Å and *R* = 5.49 Å shown in Figure 5(a), it can be observed that the number of coefficients with a relative population at or near one is higher. The increase in coefficients with high population is consistent with the emergence of static correlation in the system.^115^

With some familiarity with the relevant features contained in a state histogram, we will now explore the behavior for the density matrix histogram. Figure 5(a) shows two sets of lines, one set from ft-FCI and the other set from PIP-DMQMC, which correspond to the histogram for the entirety of the density matrix. Both sets of lines generally show good agreement, suggesting that the PIP-DMQMC method is accurately sampling the density matrix for these temperatures and systems. For the smallest bond length, there is a single matrix element with a relative population of one, and thereafter the relative population quickly falls roughly an order of magnitude. Whereas for larger bond lengths, the number of matrix elements with a large relative population (close to one) has grown considerably, which as mentioned previously for the ground state FCI wave function is consistent with greater static correlation entering the system. The observed increase in high population matrix elements for longer bonds corresponds to an increase in the cost to run DMQMC. This is because the number of data points that must be accumulated from the density matrix has increased.^103^ A similar behavior was observed in FCIQMC, though it was also found that some systems show a simultaneous compression of the wave function resulting in a net decrease for the computational cost.^115^ The compression for the wave function representation is observed in the maximum number of coefficients decreasing as the bond length increases. This behavior can be observed in Figure 5(a) for the ground state FCI wave function, as well as the density matrix, when comparing the two largest bond lengths.

Now that we have a means of reading the density matrix histogram to extract useful information, we will explore the density matrix histogram across temperatures using a larger basis set (cc-pVDZ) shown in Figure 5(b). For the equilibrium bond distance in the cc-pVDZ basis set, we find that the total number of matrix elements in the histogram has increased substantially from that of STO-3G in Figure 5(a), which is expected. This is true regardless of temperature and is primarily the result of the much larger density matrix for the larger basis set system. The effect of temperature in the cc-pVDZ basis set is noticeably less drastic for the shorter bond length. The two lowest temperature histograms are nearly indistinguishable for the smallest bond length, while the longer bond length is observed to change between the two lowest temperatures.

Now that we have a better understanding of the behavior of density matrix histograms, a natural question tends to arise: What are some of the contributions to this behavior? In Figure 5(c) we explore the contributions from the diagonal of the density matrix, as well as the matrix elements which have an excitation of two or less connecting their site labels (*n*_ex_ = 2), for the N_2_/cc-pVDZ density matrix at *T* = 63,200 K. The shape of the diagonal elements histogram appears to provide a great deal of useful information. The shorter bond length has a small number of large relative population matrix elements, which quickly falls roughly an order of magnitude to a small plateau. This is not observed for the longer bond length, where there is a more gradual loss of the relative population with a number of elements at the high relative population limit. Having more elements with a high relative population is indicative of static correlation entering the system. Observing the smallest bond distance histogram for the *n*_ex_ = 2 space, the behavior is initially similar to the diagonal but slowly begins to follow the slope for the full density matrix. This blending between the diagonal and the full matrix is also seen for the longer bond length. When the histograms for the regions corresponding to the diagonal or the *n*_ex_ = 2 space coincide with the whole density matrix, it means the density matrix comes from entirely that region. Then it is interesting to see the gap between the *n*_ex_ = 2 space histogram and the full density matrix for the shorter bond length. This suggests that much of the density matrix is in regions outside the *n*_ex_ = 2 space, as the only overlap occurs at high relative populations. Surprisingly, the longer bond length has more coincidence between the *n*_ex_ = 2 space and the density matrix. This suggests the opposite behavior; that is, much of the density matrix comes from the *n*_ex_ = 2 space. This is the temperature which has one of the largest differences in entropy between these two geometries. Though what causes this discrepancy is not immediately apparent and likely warrants future investigations into the matter.

## 4 Conclusions

The finite electronic temperature free energy surface of the N_2_ molecule was investigated using finite temperature FCI and PIP-DMQMC. For a STO-3G basis, we found that increasing the electronic temperature causes the free energy well, which corresponds to the global minimum of the free energy surface, to gradually lose depth and a small free energy barrier forms between the well and the dissociation product. The free energy difference between the equilibrium geometry and dissociation limit at low temperatures increased due to an entropic contribution, which opposed a decrease in the free energy due to the internal energy. The result is longer bond distances are favored thermodynamically, i.e. longer bond distances minimize the free energy. The temperature where the dissociated product has a lower free energy than the equilibrium bond free energy is referred to here as the crossover temperature. We found that increasing the basis set size to cc-pVDZ caused a decrease in the crossover temperature. We also explored the origins for some of these behaviors using the histogram of the density matrix. We found that the density matrix for longer bond lengths tends to respond more to changes in temperature.

While the results here are motivating for continued exploration of finite electronic temperature in less traditional contexts, there are several important steps to consider before this can be realized. Limitations of this specific study are investigations of the system dependence and how the combined effects for the basis set size and symmetries affect the crossover temperature. Methodologically, there is a need to reduce the cost for running high accuracy methods like DMQMC, as well as compare to deterministic methods within a finite temperature formulation, such as coupled cluster theory. Lastly, there is a need for a better understanding of the relevant laboratory or environmental conditions needed to observe behavior demonstrated here as well as the importance of such phenomena. With so many steps required before the physical outcomes can be explored in experiment, it can seem daunting to pursue these investigations; however, we hope the interesting behavior observed here will promote continued investigations for these phenomena so that chemical understanding may be enriched further.

## Data Availability

The data that supports the findings of this study are available within the article. For the purposes of providing information about the calculations used, files will be deposited with Iowa Research Online (IRO) with a reference number 10.25820/data.006648.

## Supporting Information Available

Text and Figures S1–S4 which describe the impact of spin polarization; the relative importance of electronic correlation in different temperature regimes; contributions to the total free energy from rotation and translation; and the complete basis set limit (PDF)
