# Supplementary material for: Electronic Free Energy Surface of the Nitrogen Dimer Using First-Principles Finite Temperature Electronic Structure Methods
Source: J Phys Chem A. 2023 Aug 3;127(32):6842–56. doi: 10.1021/acs.jpca.3c01741 (PMC10440793; doi:10.1021/acs.jpca.3c01741)
Supplement: Supplementary file 1 — jp3c01741_si_001.pdf [file jp3c01741_si_001.pdf]

Supporting Information for: "The electronic free energy surface of the nitrogen dimer using first-principles finite temperature electronic structure methods"

William Z. Van Benschoten,[a)] Hayley R. Petras,[a)] and James J. Shepherd[b)]
Department of Chemistry, University of Iowa

(Dated: August 2, 2023)

I. THE IMPACT OF SPIN POLARIZATION

In Figure 1 of the main manuscript we only included Slater determinants with $m_S = 0$ in our many-body basis. To test the sensitivity of our qualitative observations given our somewhat arbitrary choice of m_S to include, we repeated our calculation of the thermodynamic quantities initially discussed in the manuscript including all possible m_S. To remain consistent with Fig. 1 in the manuscript, we include all spatial symmetries for a given m_S. This amounts to using the same many-body basis labeled "all spin and spatial symmetries" found in the Figure 3 of the manuscript though we did not explicitly sample the spatial symmetries here. Figure S1 shows the internal energy, entropic contribution, and free energy calculated by including all possible spin polarizations. It can be observed that generally all features we observed previously are still present.

Figure S1(a) shows a decrease in the minimum of the internal energy for smaller temperatures above $T = 0$ K. For intermediate temperatures the minimum of the internal energy increases. Eventually at the highest temperatures a new minimum is found at longer bond lengths. The entropic contribution to the free energy difference in Figure S1(b) approaches zero at the high and low temperature limits. In addition, the entropic contribution is positive for smaller bond lengths, and eventually becomes negative for longer bond lengths. Finally, Figure S1(c) shows the same increase in the free energy for larger temperatures, and there is a second minimum found at longer bond lengths for the largest temperatures. This suggests that our observations for N_2/STO-3G are generally similar between the many-body basis for $m_S = 0$ or all m_S.

In addition to the qualitative consistency between the data shown here and in the manuscript, there are several noticeable changes. Including more spin polarizations resulted in the internal energy minimum decrease and the negative entropic contribution for intermediate bond lengths to generally have smaller changes in magnitude between temperature. As well, the second minimum for the largest temperatures appears smaller in magnitude. Finally, the free energy minimum tends to increase more between the smallest temperatures.

[a)]These authors contributed equally to this project
[b)]Electronic mail: james-shepherd@uiowa.edu

II. THE RELATIVE IMPORTANCE OF ELECTRONIC CORRELATION IN DIFFERENT TEMPERATURE REGIMES

Throughout the manuscript we have focused on data from methods that treat the electronic correlation problem exactly. As such, we have made no comparisons to mean-field methods which treat the electronic correlation in an approximate way. The lack of comparison is primarily due to the importance of electronic correlation at the ground state for dissociating molecules.[1,2] It follows that the temperatures close to $T = 0$ will have a similar dependence on the accuracy of the electronic correlation treatment. However, in the high temperature limit it is found that approximate methods can agree with exact methods such as ft-FCI.[3] Here we will use finite temperature unrestricted Hartree–Fock (ft-UHF) calculations to test this agreement. We compare the two methods to firstly check our assumption that an exact treatment of electronic correlation is important for low temperatures. Secondly, we determine an approximate range of temperatures where the exact treatment of correlation is important for the accurate estimates in N_2/STO-3G.

Figure S2 shows data from ft-UHF[4–7], as implemented in the PySCF code[7], and ft-FCI[3] in the grand canonical ensemble (GCE). We have switched to working in the GCE because it is implicit within ft-UHF, and generalizing our ft-FCI implementation over particle numbers was relatively straight forward, compared to converting the ft-UHF implementation we used to the canonical ensemble.[3,8,9] In the grand canonical ensemble, the many-body basis consists of determinants for all possible particle numbers N, spin polarizations m_S, and spatial symmetries.[3] Our investigation here is to compare the mean-field and exact methods behavior only. To do so we are calculating the same quantities as in Figure S1 for consistency to the manuscript. A discussion of variationality in the grand canonical ensemble is beyond the scope of this study.

In Figure S2(a), the internal energies for both methods are in qualitative disagreement for all temperatures but the highest used T used. This suggests an accurate treatment of the electronic correlation is important for the N_2/STO-3G internal energy at temperatures as high as $T = 100.0$ E_h. For the entropic contribution shown in Figure S2(b), the discrepancy between the two methods is clear for all temperatures shown. However, the two methods appear to be converging to the same curve

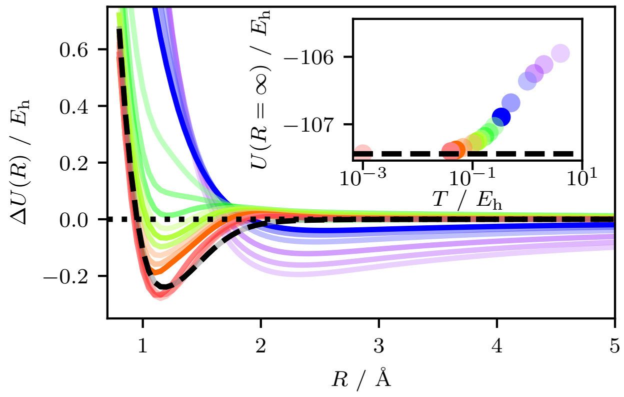

S1(a) Internal energy

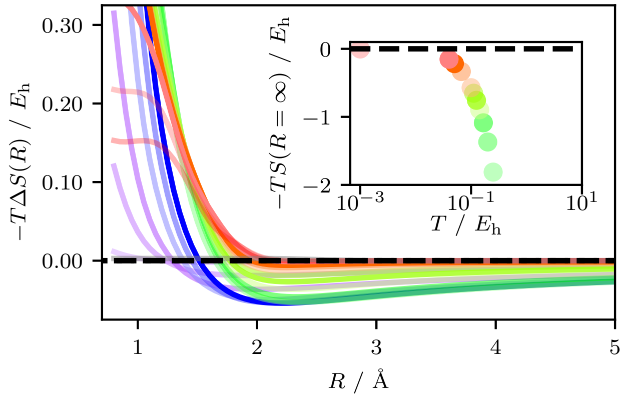

S1(b) Entropic contribution to the energy

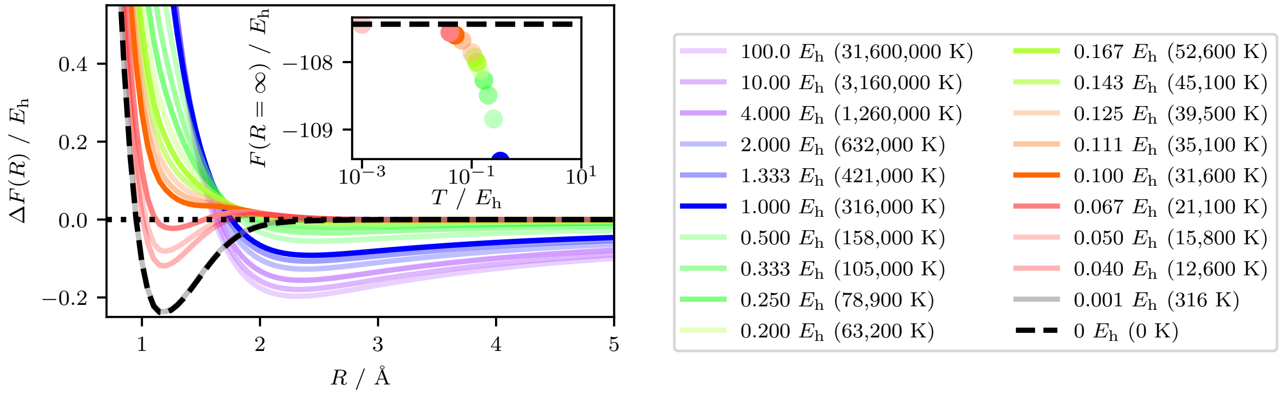

S1(c) Free energy

Figure S1. Analytical thermodynamic quantities as a function of bond distance over a range of temperatures for the N_2/STO-3G system. All thermodynamic quantities are calculated by including all spin polarizations m_s and spatial symmetries for the N_2 dimer in the many-body basis. The many-body basis used here is the same as the data labeled as 'All spin and spatial' shown in the manuscript. The (a) internal energy and (b) negative of the thermally scaled entropy are added together to calculate (c) the free energy. Each temperature curve has the largest bond distance value subtracted thereby setting the largest bond distance to zero. The values subtracted from each curve are shown in the inset for a small energy range near the ground state ($T = 0$ K). To aid in interpretation the data presented using a similar format found within the manuscript.

at higher temperatures, as suggested by the similarity between the highest temperature entropic contributions. Combining the two quantities generates the free energy shown in the last figure (Figure S2(c)), which largely appears similar to the internal energy curve. That is to say, the highest temperature shows agreement between the two methods, while the lower temperatures are in visual disagreement. This makes sense considering the magnitude of the internal energy is generally larger than the entropic contributions. Overall, this suggests that the exact treatment of electronic correlation is important in the internal energy, entropy, and free energy for the N_2/STO-3G system for temperatures as high as $T = 100.0\ E_h$. We believe that the crossover temperature will reflect similar deviations found between ft-UHF and ft-FCI in the quantities discussed here. Lastly, we note that our previous investigations[9] on the specific heat capacity and entropy suggest that the accurate treatment of electronic correlation is most important in these same quantities for $T < 1.0\ E_h$, corresponding to the lowest-T peak in the specific heat capacity. This effect is likely dependent on basis set.

III. CONTRIBUTIONS TO THE TOTAL FREE ENERGY FROM ROTATION AND TRANSLATION

Practical applications using the internal partition function of diatomic and small polyatomic molecules often use a separable form of the partition function.[10] The total partition function is given as:

$$Z_{\mathrm{tot}} = Z_{\mathrm{int}} Z_{\mathrm{trans}}, \tag{S1}$$

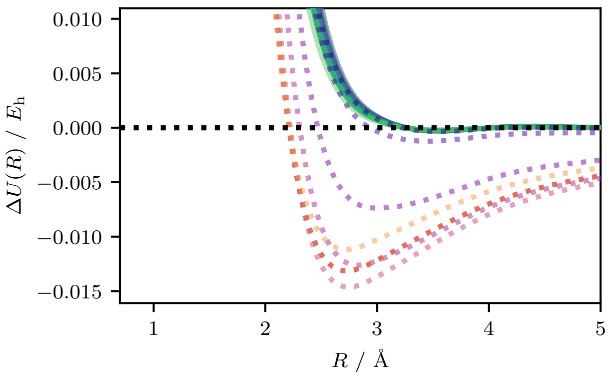

S2(a) Internal energy

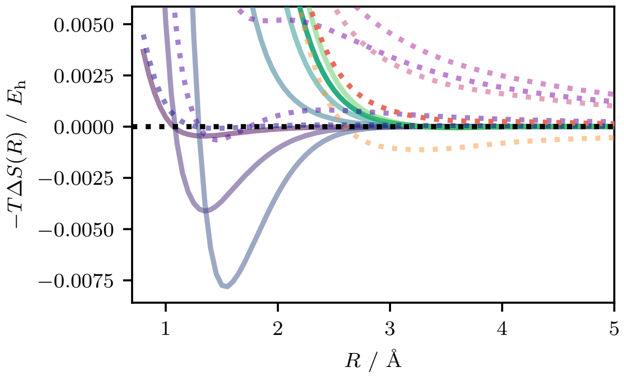

S2(b) Entropic contribution to the free energy

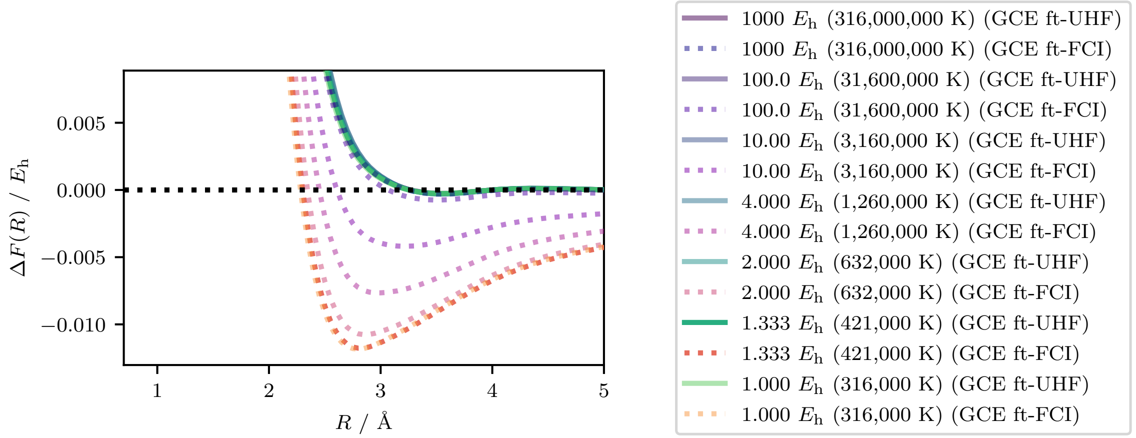

S2(c) Free energy

Figure S2. Analytical thermodynamic quantities as a function of bond distance over a range of temperatures for the N_2/STO-3G system. Both the ft-UHF and ft-FCI data are calculated in the grand canonical ensemble using $N = 14$ total electrons. The (a) internal energy and (b) negative of the thermally scaled entropy are added together to calculate (c) the free energy. Each temperature curve has the largest bond distance value subtracted thereby setting the largest bond distance to zero. To aid in interpretation the data shown here are presented in a similar way for figures in the manuscript.

then the internal partition function is further separated into different contributions:

$$Z_\mathrm{int} = Z_\mathrm{vib} Z_\mathrm{rot} Z_\mathrm{el}. \tag{S2}$$

For this study, we have generally considered Z_el as a function of R, the internuclear coordinate which is also the coordinate associated with the vibrational degrees of freedom. Thus, a $Z(R)$ can have contributions from Z_trans and Z_rot respectively. The partition functions for the translational and rotational degrees of freedom are calculated as set out below using textbook formulae. The equations are presented assuming quantities expressed in Hartree atomic units. The free energy contributions are then calculated using expresions from the manuscript.

We have calculated the partition function for the ideal rigid rotor as

$$Z_\mathrm{rot} = \frac{1}{\sigma} \sum_J (2J+1) e^{-\beta 2\pi B J(J+1)}, \tag{S3}$$

where σ is the symmetry factor, the $(2J+1)$ quantity represents the degeneracy of the Jth eigenstate, and B is the rotational constant. The quantity σ is equal to 2 for homonuclear diatomics (assuming $^{14}N_2$). The rotational constant is given by $B = \frac{1}{4\pi R^2} \frac{m_1+m_2}{m_1 m_2}$ where m_1 and m_2 are equal to the mass of the nitrogen atom. To numerically approximate the infinite summation for the rotational partition function the number of states J included in the summation was increased in steps of 1000 until the partition function remain fixed to the twelfth decimal place.

The translational partition function for an ideal gas was approximated as

$$Z_\mathrm{trans} = V \left[\frac{(m_1+m_2)T}{2\pi} \right]^{3/2}, \tag{S4}$$

where Hartree atomic units make $\hbar = 1$. We calculated the volume, V, by using one mole of an ideal gas at stan-

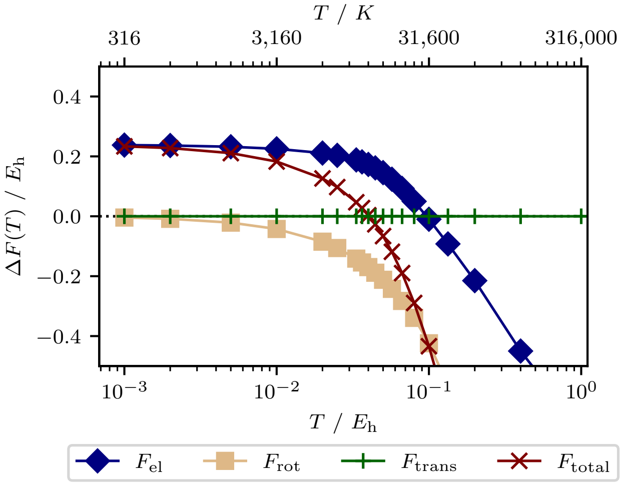

Figure S3. The free energy difference to the 1.2 Å separated N_2 dimer for several degrees of freedom, along with the corresponding total free energy, for the $R = 10.0$ Å N_2/STO-3G dimer as a function of temperature. The contributions for the electronic free energy (F_{el}) are calculated using the many-body basis containing determinants for $m_S = 0$ that conserve the particle number. The rotational contribution (F_{rot}) is calculated using the ideal rigid rotor approximation and the translation contribution (F_{trans}) is calculated assuming behavior like an ideal gas.

dard pressure and temperature.

We again examine the effects of adding translational and rotational degrees of freedom from the viewpoint of the crossover temperature where the dissociation limit becomes thermodynamically favored. Figure S3 shows the free energy difference to the 10.0 Å N_2 dimer for a single bond length ($R = 1.2$ Å) of N_2/STO-3G. We used 10.0 Å because we wanted to include the effect of rotation and the rigid rotor approximation would break down at large separations. Modelling the dissociation explicitly as nitrogen atoms makes the partition function expressions more complicated (because of the dimension of the relevant Hilbert spaces) and we felt like this was beyond the scope of this manuscript. This choice has the effect that the translational partition function is the same and contributes no change in free energy. For the rotational contributions, for the temperatures shown the internal energy is equal for both bond lengths due to the equipartition theorem. However, the rotational entropy makes a positive impact on the free energy difference and means that the stretched/dissociated bond length becomes more thermodynamically stable at a lower temperature ($\sim$ 12,600 K) as compared not including rotation.

We note in passing that, when treating Z_{el} in the context of other degrees of freedom, it is common for some authors to use energy cutoffs in the range of 10,000s of wavenumbers[11,12]. This is context specific and when applied to the spectroscopic line lists it has been shown that this energy cutoff does not affect accuracy. However, we do not apply an explicit cutoff here.

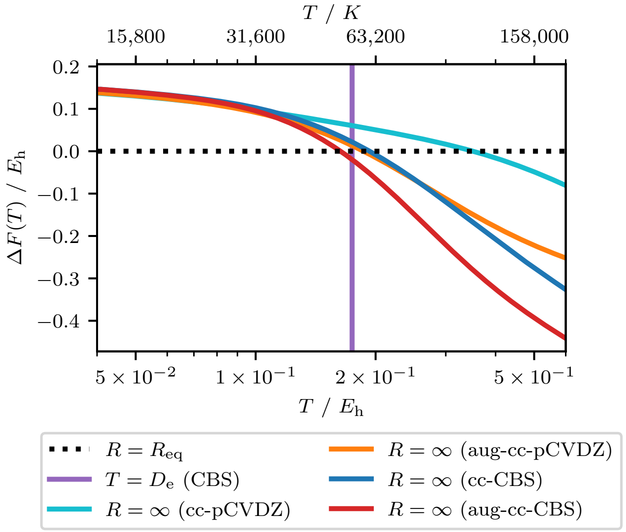

Figure S4. The free energy difference to the 0.74 Å separated H_2 dimer ($R = R_{\mathrm{eq}}$) as a function of temperature for the H_2 system using two basis sets and two estimates for the complete basis set limit. Here $R = \infty$ refers to the largest bond distance in our data set ($R = 200$ Å). The symmetries included are all those spatial symmetries for $m_S = 0$ and $N = 2$. The calculated dissociation energy D_e for the complete basis set limit corresponds to where both the complete basis set limit estimates where found to agree, e.g. between the correlation consistent and augmented correlation consistent basis sets, at $T = 0$.

IV. THE COMPLETE BASIS SET LIMIT

In this section we consider broadly how some of the qualitative observations in this manuscript may change when extrapolating our calculated thermodynamic quantities to the complete basis set limit (CBS), as well as discussing issues related to the divergence of the electronic partition function. The impact that using a finite basis set has not been explored in detail beyond the electron gas for finite temperature electronic structure.[9,13] As pointed out by Malone et al., the $T = 0$ limit serves as a good estimate for the limiting behavior.[14] Here we will follow a similar approach and use $T = 0$ methods for extrapolating to the complete basis set when performing calculations on molecules. We wish to emphasize that this may become increasingly inaccurate at higher and higher T and further study is required. Note that such methods to complete the basis in a hydrogen atom could encounter similar problems as the divergence in the electronic partition function that has been analytically shown for an exact treatment of an isolated hydrogen atom.[15–17] While we investigated this possibility, we did not find any evidence of this for H_2.

To perform the extrapolations to the complete basis set limit we first separate out the Hartree–Fock-like contribution to the energy from the correlation-like contribution. This follows a similar procedure for extrapolating the internal energy in Hartree–Fock.[18,19] For the Hartree–

Fock-like energy, we use a thermal sum over the diagonal of the Hamiltonian matrix in the HF basis[20]. This is referred to as THF. Then the equation used to extrapolate THF is:

$$F^{(\mathrm{THF})}(X) = F^{(\mathrm{THF})}(\mathrm{CBS}) + F^{(\mathrm{THF})}e^{-CX}, \quad \text{(S5)}$$

where X is the cardinality for a given Dunning basis set, e.g. cc-pVDZ has a cardinality of $X = 2$.[21] Following the method for fitting the internal correlation energy at $T = 0$[22], we fit the difference between the ft-FCI free energy and the THF free energy as

$$\delta F(X) = \delta F(\mathrm{CBS}) + AX^{-3}, \quad \text{(S6)}$$

where $\delta F(X) = F^{(\mathrm{FCI})}(X) - F^{(\mathrm{THF})}(X)$. To estimate the complete basis set, we first fit the $F^{(\mathrm{THF})}(X)$ using the cc-pCV5Z ($X = 5$) and cc-pCV6Z ($X = 6$) basis sets as implemented in Molpro[23] as well as the cc-pV7Z ($X = 7$) basis set[24]. We also used the augmented version of the same basis sets, e.g. aug-cc-pCV5Z and aug-cc-pCV6Z as given in Molpro and aug-cc-pV7Z[24,25]. We note that the (aug-)cc-pCVXZ and (aug-)cc-pVXZ basis sets are equivalent for the H_2 system we used for this investigation, and the naming convention is kept for consistency with our input files to Molpro. Equation S5 was fit using $X = 5$, $X = 6$, and $X = 7$, and Eq. (S6) was fit with only $X = 6$ and $X = 7$. For comparisons, we also calculated data for the cc-pCVDZ and aug-cc-pCVDZ basis sets as implemented in Molpro. To calculate the CBS, we combined the extrapolations from Eq. (S5) with the extrapolations from Eq. (S6).

Figure S4 shows the free energy difference to the $R = 0.74$ Å geometry for the double-ζ basis sets, as well as the complete basis set limits from both types of basis set. Here we observe that at low temperature all the basis sets are in reasonable agreement, though the two double-ζ basis sets can be seen to fall slightly below the CBS estimates. At higher T, there are noticeable differences in the behaviors of each estimate. The cc-pCVDZ basis has a more gradual decrease in the free energy difference to the dissociated product compared to the other estimates. The result is that the crossover in the free energy, where the free energy is lower for the dissociated product, occurs at a higher temperature compared to the other curves. Additionally, the crossover is much greater than the estimated dissociation energy relative to the other crossovers. The cc-CBS estimate results in minimal changes at low temperature, but substantial changes in the free energy difference at higher temperature. The result is the crossover occurs at a lower T, much closer to the calculated zero temperature dissociation energy. A similar, but less pronounced, behavior can be observed in the augmented versions of the basis sets. Interestingly, the aug-cc-CBS crossover temperature is below the dissociation energy, where all other estimates were above.

It is worth re-iterating that an important issue in completing the basis set via extrapolating the cardinal number to infinity is that the electronic partition function of an isolated system is known to diverge when summing over all cardinal numbers. This is a complicated subject that has long been noted in the astrochemistry/astrophysics literature evaluating electronic states.[11,26] In realistic situations, surrounding atoms change this picture and this removes the (unphysical) divergence. There are many phenomenological descriptions for how this occurs and options for its removal during modelling.[11,26] In our case, the lack of evidence of a divergence can originate from a wide range of sources. Two that we can think of are (1) not including a high enough cardinal number in our basis, and (2) that the basis functions themselves are optimized for ground state calculations.

REFERENCES

[1] Dutta, A.; Sherrill, C. D. Full configuration interaction potential energy curves for breaking bonds to hydrogen: An assessment of single-reference correlation methods. *J. Chem. Phys.* **2003**, *118*, 1610–1619.

[2] Bauschlicher, C. W.; Langhoff, S. R.; Taylor, P. R.; Handy, N. C.; Knowles, P. J. Benchmark full configuration-interaction calculations on HF and NH_2. *J. Chem. Phys.* **1986**, *85*, 1469–1474.

[3] Kou, Z.; Hirata, S. Finite-temperature full configuration interaction. *Theor. Chem. Acc.* **2014**, *133*, 1487.

[4] Mermin, N. Stability of the thermal Hartree-Fock approximation. *Annals of Physics* **1963**, *21*, 99–121.

[5] Mermin, N. D. Thermal Properties of the Inhomogeneous Electron Gas. *Phys. Rev.* **1965**, *137*, A1441–A1443.

[6] Stoitsov, M. V.; Petkov, I. Z. Density functional theory at finite temperatures. *Annals of Physics* **1988**, *184*, 121–147.

[7] Sun, Q. et al. Recent developments in the PySCF program package. *J. Chem. Phys.* **2020**, *153*, 024109.

[8] Hirata, S. Finite-temperature many-body perturbation theory for electrons: Algebraic recursive definitions, second-quantized derivation, linked-diagram theorem, general-order algorithms, and grand canonical and canonical ensembles. *J. Chem. Phys.* **2021**, *155*, 094106.

[9] Van Benschoten, W. Z.; Weiler, L.; Smith, G. J.; Man, S.; DeMello, T.; Shepherd, J. J. Electronic specific heat capacities and entropies from density matrix quantum Monte Carlo using Gaussian process regression to find gradients of noisy data. *J. Chem. Phys.* **2023**, *158*.

[10] McQuarrie, D.; Simon, J. *Physical Chemistry: A Molecular Approach*; G - Reference,Information and Interdisciplinary Subjects Series; University Science Books, 1997.

[11] Barklem, P. S.; Collet, R. Partition functions and equilibrium constants for diatomic molecules and atoms of astrophysical interest. *Astron. Astrophys.* **2016**, *588*, A96.

[12] Yurchenko, S. N.; Szabó, I.; Pyatenko, E.; Tennyson, J. ExoMol line lists XXXI: spectroscopy of lowest eights electronic states of C2. *Mon. Notices Royal Astron. Soc.* **2018**, *480*, 3397–3411.

[13] Malone, F. D.; Blunt, N. S.; Shepherd, J. J.; Lee, D. K. K.; Spencer, J. S.; Foulkes, W. M. C. Interaction picture density matrix quantum Monte Carlo. *J. Chem. Phys.* **2015**, *143*, 044116.

[14] Malone, F. D. Quantum Monte Carlo Simulations of Warm Dense Matter. Ph.D. thesis, Imperial College London, 2017.

[15] Strickler, S. J. Electronic partition function paradox. *J. Chem. Educ.* **1966**, *43*, 364.

[16] Plastino, A.; Rocca, M.; Ferri, G. Resolving the partition function's paradox of the hydrogen atom. *Physica A* **2019**, *534*, 122169.

[17] Blinder, S. M. Canonical partition function for the hydrogen atom via the Coulomb propagator. *J. Math. Phys.* **1995**, *36*, 1208–1216.

[18] Peterson, K. A.; Kendall, R. A.; Dunning, T. H. Benchmark

calculations with correlated molecular wave functions. III. Configuration interaction calculations on first row homonuclear diatomics. *J. Chem. Phys.* **1993**, *99*, 9790–9805.

[19] Jensen, F. The basis set convergence of the Hartree–Fock energy for H2. *J. Chem. Phys.* **1999**, *110*, 6601–6605.

[20] Malone, F. D.; Blunt, N.; Brown, E. W.; Lee, D.; Spencer, J.; Foulkes, W.; Shepherd, J. J. Accurate Exchange-Correlation Energies for the Warm Dense Electron Gas. *Phys. Rev. Lett.* **2016**, *117*, 115701.

[21] Dunning, T. H. Gaussian basis sets for use in correlated molecular calculations. I. The atoms boron through neon and hydrogen. *J. Chem. Phys.* **1989**, *90*, 1007–1023.

[22] Halkier, A.; Helgaker, T.; Jørgensen, P.; Klopper, W.; Olsen, J. Basis-set convergence of the energy in molecular Hartree–Fock calculations. *Chem. Phys. Lett.* **1999**, *302*, 437–446.

[23] Werner, H.-J.; Knowles, P. J.; Knizia, G.; Manby, F. R.; Schütz, M.; others MOLPRO, 2019.2 , a package of ab initio programs. 2019; see https://www.molpro.net.

[24] Feller, D.; Peterson, K. A. Probing the limits of accuracy in electronic structure calculations: Is theory capable of results uniformly better than "chemical accuracy"? *J. Chem. Phys.* **2007**, *126*, 114105.

[25] Feller, D. Application of a convergent, composite coupled cluster approach to bound state, adiabatic electron affinities in atoms and small molecules. *J. Chem. Phys.* **2016**, *144*, 014105.

[26] Hummer, D. G.; Mihalas, D. The equation of state for stellar envelopes. I - an occupation probability formalism for the truncation of internal partition functions. *Astrophys. J.* **1988**, *331*, 794.
